# Psychological Distress in a Sample of Inpatients With Mixed Cancer—A Cross-Sectional Study of Routine Clinical Data

**DOI:** 10.3389/fpsyg.2020.591771

**Published:** 2020-11-30

**Authors:** Luisa Peters, Jan Brederecke, Anke Franzke, Martina de Zwaan, Tanja Zimmermann

**Affiliations:** ^1^Department of Psychosomatic Medicine and Psychotherapy, Hannover Medical School, Hanover, Germany; ^2^Department of Hematology and Oncology, Hannover Medical School, Hanover, Germany

**Keywords:** cancer, distress, psychosocial, quality of life, distress thermometer

## Abstract

**Background:**

The diagnosis and treatment of cancer are associated with psychological distress that often leads to a significant reduction in emotional and physical well-being and quality of life. Early detection of psychological distress is therefore important. This study aims to assess the psychological distress of inpatient cancer patients using routine clinical data. Furthermore, variables and problems most strongly associated with psychological distress should be identified.

**Materials and Methods:**

*N* = 1,869 inpatients were investigated (mean age = 60.89 years; 35.94% female) using the National Comprehensive Cancer Network Distress Thermometer and problem checklist to assess distress as well as multiple possible problem areas. Visceral oncological cancer (31.6%) was the most common tumor diagnosis, followed by skin cancer (26.2%) and urological cancer (21.7%).

**Results:**

65.9% of the sample experienced high levels of distress (Distress Thermometer ≥ 5). Female sex, stage 4 of disease, and visceral and head and neck cancer emerged as risk factors for high distress. A younger age (<65 years) was significantly correlated with higher distress. The most frequently self-reported problems were fears (50.1%), worry (49.9%), and fatigue (49.1%). Patients with all 3 of these problems had 24 times higher risk [odds ratio (OR) = 23.9] for high levels of distress than patients without these problems. Women reported significantly more practical, emotional, and physical problems than men. Younger (<50 years) and middle-aged patients (50–64 years) reported increased levels of practical, family, and emotional problems compared with older patients (≥65 years).

**Discussion:**

Almost two-thirds of the sample reported high levels of distress. The most frequently reported problem areas were emotional and physical problems. These results can help to identify patients with high risk for psychological distress and, therefore, be used to optimize psychosocial and psycho-oncological care for patients with cancer.

## Introduction

The incidence of cancer continues to increase worldwide, with 18.1 million cases per year (Bray et al., 2018). At the same time, mortality is decreasing due to improvements in medical treatment and early detection, which improves the life expectancy and survival rate of cancer patients (Siegel et al., 2019). The diagnosis and treatment of cancer remain a major stressful life event that can lead to high levels of psychological distress (Meggiolaro et al., 2016; Kim et al., 2017; Mehnert et al., 2018). Distress in cancer patients is associated with reduced quality of life and functional status (Kendall et al., 2011), lower treatment adherence, and pain (Brown et al., 2003; Kim et al., 2017). Also, the risk of developing mental disorders increases (Mehnert et al., 2014). The most common comorbid mental disorders are adjustment disorders (13%), followed by depression (11%), and anxiety disorders (10%) (Mehnert, 2014).

According to the National Comprehensive Cancer Network (NCCN), cancer-related distress can be defined as “a multidetermined unpleasant emotional experience of a psychological, social, and/or spiritual nature that may interfere with the ability to cope effectively with cancer, its physical symptoms, and its treatment” (p. 6; National Comprehensive Cancer Network [NCCN], 2020b). Approximately 50% of cancer patients report clinical levels of distress (Mehnert et al., 2018). The concordance between the distress ratings of patients and physicians is low (Werner et al., 2012). Therefore, to identify those patients with high distress, screening for distress is necessary (Holland, 2013) and recommended by the NCCN.

In particular, validated screening tools are recommended to screen patients for distress in everyday clinical practice (Pirl et al., 2014) and to improve communication and recommendations for psychosocial support (Mitchell, 2013). Several instruments have been proposed, including very short questionnaires such as the Distress Thermometer (DT; Nationale Comprehensive Cancer Network [NCCN], 2004). The DT is a simple and rapid self-reported tool to effectively screen for distress symptoms using a 0–10 rating scale. In addition, a problem list helps to identify sources of distress. The DT has demonstrated good reliability and is available in numerous languages (Ownby, 2019) as well as in German (Mehnert et al., 2006). The advantages are the brevity, the simple application, and the easy filling of the DT. As a limitation, the DT does not capture aspects of distress related to suicidal ideation (i.e., hopelessness), isolation, or loneliness. Moreover, the religious and spiritual section is very brief and does not capture the concerns of those with different faith and from varied ethnic backgrounds. Other tools, such as the Holistic Needs Assessment (National Cancer Action Team, 2011), to detect psychological distress among cancer patients take these aspects more closely into account.

Systematic and valid screening for distress helps identify patients with elevated levels of distress and those who might benefit from further psychosocial or psycho-oncological support or interventions (Feldstain et al., 2014). More accurate referrals to psychosocial services and improved communication between patients and clinicians can be ensured through routine distress screening (Carlson et al., 2019). Moreover, interventions to reduce distress were shown to be effective in reducing the symptom burden among cancer patients. In addition, a positive impact on families, cancer outcomes, and the medical system can be observed when distress is addressed (Ehlers et al., 2019). These findings highlight the importance of routine screening for distress, followed by individualized psycho-oncological care when required (Hamilton and Kroska, 2018).

Previous studies still show ambiguous results regarding the prevalence of distress, sex and age differences, and other demographic data as well as between different types of cancer. The prevalence of distress among cancer patients ranges from 30% (Zabora et al., 2001; Kendall et al., 2011) to 60% (Meggiolaro et al., 2016). Studies differentiate between inpatients (patients who are in hospital treatment) and outpatients (patients who are in outpatient or no medical treatment). In most studies, inpatients show higher distress than outpatients, e.g., 37.8% of clinically distressed patients in cancer outpatients (Carlson et al., 2004) vs. 63.5% in cancer inpatients (Clark et al., 2011). A large multicenter study in the United States and Canada by Carlson et al. (2019) identified 46.2% of cancer patients being clinically significantly distressed. Another recent study conducted with a large German sample of mixed cancer in- and outpatients showed 52% of the sample experiencing significant distress (Mehnert et al., 2018).

Regarding sex differences, studies show higher distress among female cancer patients than male patients (Meggiolaro et al., 2016; Kim et al., 2017; Hamilton and Kroska, 2018; Mehnert et al., 2018). Especially regarding emotional distress, women seem to show a significantly higher burden with twice as likely to experience depressive and anxiety symptoms than men in a British cancer sample (Linden et al., 2012).

Regarding age, Mehnert et al. (2018) identified patients aged 60 years or older as having the highest levels of distress in a German cancer sample. The majority of studies, however, could identify a younger age as a risk factor for reporting higher levels of distress (Zabora et al., 2001; Giese-Davis et al., 2012; Hamilton and Kroska, 2018). Gao et al. (2010) found that every 1-year increase in age was associated with a 3% reduced risk of distress in a UK sample. Carlson et al. (2019) found that US patients between 30 and 69 years were more distressed than patients between 70 and 79 years and those older than 80 years. Patients between 40 and 49 years were 2.3 times more likely to experience distress than patients older than 79 years.

Additionally, unemployment and lower educational level emerged as further risk factors for high distress, whereas being married seems to be a protective factor (Zabora et al., 2001; Giese-Davis et al., 2012). Moreover, patients with an advanced stage of the disease show a higher risk for distress (Kim et al., 2017; Mehnert et al., 2018).

The association between cancer type and distress seems less clear. Mehnert et al. (2018) indicate that patients with cancer of the female genital organs or pancreatic cancer experience the highest levels of distress. Krebs et al. (2018) support these findings, whereas Zabora et al. (2001) and Carlson et al. (2004) found lung cancer patients to have the highest risk for distress and patients with gynecological or breast cancer to have the lowest rates of distress. Carlson et al. (2019) as well found patients with pancreatic and lung cancer to be more likely to be distressed, whereas those with gynecological and prostate cancer were less likely to be distressed. Lavelle et al. (2017), on the other hand, found no effect of cancer type on distress. In a large sample of more than 10,000 cancer patients in the United Kingdom, gynecological, hematological, and lung cancer patients reported the highest levels of emotional distress (symptoms of depression and anxiety). Patients with skin and prostate cancer reported lower levels of emotional distress than the average sample (Linden et al., 2012).

Herschbach et al. (2019) investigated psychological distress in a large German database with almost 20,000 cancer patients. They could identify a cluster of minimally distressed patients, who consists of more men, older patients, more prostate cancer, and fewer breast cancer patients. A cluster of highly distressed patients was found consisting of patients with the advanced disease from acute care hospitals or outpatients and gynecological, respiratory, upper gastrointestinal, urinary, hematologic, testicular, and ear/neck/throat cancer, as well as neuro-oncologic tumors. Moreover, three further clusters of patients showed a medium distress level and consisted of mainly physically distressed patients, mainly psychologically distressed, and mainly socially distressed patients. They differed with regard to age, sex, cancer type, and treatment setting (Herschbach et al., 2019). These results show that despite a large heterogeneity in the experience of distress, sociodemographic and disease-related variables are associated with a high risk for distress.

In addition to the extent of psychological distress, the areas in which patients experience stress are also important. Physical, practical, and emotional problems are frequent in cancer patients and can predict clinical levels of psychological distress (Giese-Davis et al., 2012). Especially emotional problems are often reported and strongly associated with distress (Kendall et al., 2011; Blenkiron et al., 2014). Worry (negative thoughts or images, mostly uncontrollable) seems like the most prevalent emotional problem in cancer patients (Kendall et al., 2011; Blenkiron et al., 2014). Mehnert et al. (2018) could identify 46.9% of cancer patients reporting worry as the most frequently experienced emotional problem, with females reporting even 52.1% of worries. Another very commonly reported emotional concern is fear(s) (emotion induced by perceived danger or threat that causes physiological and behavioral changes). In the German sample of Mehnert et al. (2018), fears were the second common emotional problem with a prevalence of 42%. Tonsing and Vungkhanching (2018) found fears to be experienced at the same high level as worry (both 40.9%) by cancer patients.

In addition to emotional problems, cancer patients also frequently report physical problems that are strongly associated with distress as well (Blenkiron et al., 2014). In the study of Guan et al. (2019), physical problems were reported as the major source of distress experienced by 93% of advanced cancer inpatients, followed by emotional problems reported by 69.2%. Pain is one of the most common symptoms experienced by cancer patients, and it has a severe impact on the patient’s well-being and quality of life (van den Beuken-van Everdingen et al., 2016; Guan et al., 2019). Another high frequently reported symptom by cancer patients is fatigue, which can be defined as “a distressing persistent subjective sense of physical, emotional, and/or cognitive tiredness or exhaustion related to cancer or cancer treatment that is not proportional to recent activity and that interferes with usual functioning” (p. 5; National Comprehensive Cancer Network [NCCN], 2020a). The prevalence of fatigue in cancer patients ranges from 50 to 90% (Hofman et al., 2007; Escalante et al., 2014; Wang et al., 2014). Cancer-related fatigue is reported not only during the treatment of cancer but also by approximately 40% of patients at the time of diagnosis and by 20–50% of patients after the end of treatment (Roila et al., 2019). Moreover, fatigue can also be experienced when patients are in survivorship or remission. Fatigue has been mentioned by cancer patients to be the major obstacle to normal functioning and well-being (Theobald, 2004). Next to fatigue and pain, insomnia is also one of the most common symptoms reported by patients with cancer. The prevalence rates are from 30 to 50% and even higher in advanced-stage cancer patients (Theobald, 2004). There is a relationship between these commonly experienced problems. For example, insomnia is a significant predictor of severe fatigue in patients with cancer and contributes to its maintenance. Also, physical symptoms such as pain or nausea/vomiting and emotional distress (depression and anxiety) are significantly associated with cancer-related fatigue (Oh and Seo, 2011). It should be considered that many of these symptoms (physical, practical, and emotional) are interrelated, so there is a risk of tautology, especially because most of the studies are cross-sectional studies.

Given the high prevalence of distress and problems experienced by cancer patients, the early detection of patients with a high risk of psychological distress is mandatory. Thereby, it is important to identify risk factors for high distress on the one hand but also to screen repeatedly for psychological distress on the other hand. Moreover, identifying patients with high distress can optimize psycho-oncological care (Giese-Davis et al., 2012; Carlson, 2013). So far, most research in the field is done with samples of female breast cancer patients or mixed samples of primarily female breast cancer patients. Therefore, further studies with other tumor entities are needed to solidify the evidence found.

To generate more generalizable knowledge about distress in cancer inpatients, the present study aimed at investigating the prevalence of distress in a large sample of inpatients by using the German version of the NCCN DT including the problem checklist (Mehnert et al., 2006) as part of the routine clinical care. In contrast to the study of Mehnert et al. (2018), only inpatients were investigated, assuming that these patients may experience higher levels of distress than outpatients or patients from rehabilitative settings. Inpatients might experience higher distress because of more serious health conditions, symptom burden, and greater uncertainty regarding the course of illness resulting from hospitalization (Peters and Sellick, 2006).

To identify patients with a higher risk for distress, the associations between psychological distress and demographic variables such as sex, age, employment status, and family status as well as disease-related variables such as diagnosis and stage of disease were investigated. According to previous research findings (Giese-Davis et al., 2012; Mehnert et al., 2018; Carlson et al., 2019), it was expected that the female sex, a younger age, unemployment, and not being married or living in a relationship, as well as advanced stages of the disease, would emerge as risk factors for high distress. There was no hypothesis regarding cancer type due to different findings in the literature.

Furthermore, frequent problems and their associations with distress were identified. Former studies have shown especially emotional problems, such as worry and fears and physical problems such as fatigue, pain, and insomnia, to be prevalent in cancer patients. Therefore, it was expected to find similar patterns in this sample of cancer inpatients.

## Materials and Methods

### Participants and Procedure

According to the recommendation of the S3-Guideline of the German Cancer Society (Deutsche Krebsgesellschaft and Deutsche Krebshilfe, 2014), standardized screening for psychological distress is conducted with cancer inpatients at Hannover Medical School as routine clinical care. All cancer patients are asked to fill out the DT during their stay at the hospital since 2015. Inclusion criteria were the presence of cancer disease and sufficient knowledge of the German language to fill out the questionnaires. The survey was part of routine medical care and therefore offered to all cancer patients. Nevertheless, the completion of the questionnaires was voluntary and could be refused without giving reasons. No data are available regarding the non-responders. Along with that, sociodemographic information, including sex, date of birth, family status, employment status, and number of children, was collected. For the current study, screening data were obtained from patients diagnosed with a malignant tumor and received inpatient treatment between May 2015 and October 2019. Disease-related information including diagnosis, Union for International Cancer Control (UICC) stage, and time since diagnosis was obtained from the cancer registry of the hospital and completed with data from patients’ medical records. The study was an anonymous retrospective analysis of existing clinical routine data. In accordance with the medical professional law in Germany, this analysis was therefore not subject to consultation by an ethics committee.

### Sample Characteristics

Demographic characteristics and medical data of the sample are presented in Table 1. The sample consists of *N* = 1,869 cancer patients with *n* = 666 women (35.94%) with a mean age of 60.89 years (*SD* = 13.38). The average age of male participants (*n* = 1,187) was 64.06 years (*SD* = 12.83). The different tumor entities were classified analogous to certification criteria in Germany. Visceral cancer was the most frequent cancer type with 31.6%. This group includes patients with liver (*n* = 203), colorectal (*n* = 165), stomach (*n* = 100), and pancreatic cancer (*n* = 54), as well as gallbladder (*n* = 37) and lung cancer (*n* = 32). Second most frequent type of cancer was skin cancer (*n* = 490; 26.2%) followed by urological cancer (21.7%). This group includes patients with bladder (*n* = 178), prostate (*n* = 166), and renal cancer (*n* = 61). Head and neck cancers (*n* = 184; 10.3%) were the fourth frequent group. The group of other cancer types consists of patients with gynecologic cancer (*n* = 38), thyroid (*n* = 28), soft tissue (*n* = 18), hematological (*n* = 15), and malignant tumors of the bone (*n* = 3), brain (*n* = 3), and eye (*n* = 14). On average, patients had 1.90 diagnoses (*SD* = 1.07). The number of cancer diagnoses ranges from 1 to 7. More than 50% (*n* = 956) had a second cancer diagnosis, and 23.5% (*n* = 439) patients had a third diagnosis. Of the patients, 11.4% had four diagnoses or more.

**TABLE 1:** Demographic characteristics and medical data of the total sample (*N* = 1,869) and for males (*n* = 1,187) and females (*n* = 666).

	Total sample (*N* = 1,869)	Women (*n* = 666)	Men (*n* = 1,187)
**Mean age in years**	62.95	60.89	64.06
(SD, range)	(13.38, 19–96)	(14.07, 19–96)	(12.83, 20–93)
**Family status (*n*, %)**			
Married	1,209 (64.7)	376 (57.4)	821 (70.0)
Single	319 (17.3)	130 (19.8)	188 (16.0)
In a relationship	176 (9.5)	54 (8.2)	120 (10.2)
Widowed	140 (7.6)	95 (14.5)	44 (3.8)
**Employment status (*n*, %)**			
Retired	1,035 (56.3)	335 (51.5)	693 (59.2)
Employed	579 (31.5)	214 (32.9)	358 (30.6)
Housewife/-husband	59 (3.2)	49 (7.5)	10 (0.9)
Unemployed	43 (2.3)	14 (2.2)	29 (2.5)
Other	121 (6.6)	39 (6.0)	80 (6.9)
**Mean number of children**	1.63	1.54	1.68
(SD, range)	(1.13, 0–8)	(1.09, 0–5)	(1.14, 0–8)
**Time since diagnosis in months**	5.41	6.21	4.99
(SD, range)	(20.47, 0–293)	(21.91, 0–272)	(19.69, 0–293)
Median (IR)	0.43 (2.30)	0.60 (2.24)	0.37 (2.37)
**Cancer type (*n*, %)**			
Visceral	591 (31.6)	213 (33.6)	374 (32.6)
Skin	490 (26.2)	210 (33.2)	278 (24.2)
Urological	405 (21.7)	53 (8.4)	351 (30.5)
Head and Neck	184 (10.3)	76 (12.0)	108 (9.4)
Other	119 (6.7)	81 (12.8)	38 (3.3)
**Mean number of diagnoses**	1.90	1.85	1.93
(SD, range)	(1.07, 1–7)	(1.03, 1–7)	(1.10, 1–7)
**UICC^*a*^ Stage (*n*, %)**			
I	375 (30.0)	162 (35.2)	212 (27.0)
II	344 (27.5)	105 (22.8)	237 (30.2)
III	262 (21.0)	99 (21.5)	161 (20.5)
IV	268 (21.5)	94 (20.4)	174 (22.2)
**Psychological distress**			
DT mean (SD, range)	5.54 (2.84, 0–10)	6.22 (2.69, 0–10)	5.16 (0.86, 0–10)

**^*a*^UICC, Union for International Cancer Control. IR, interquartile range; DT, Distress Thermometer.**

### Measurements

To measure psychological distress, the German version of the NCCN DT (Mehnert et al., 2006) was used. The DT is a screening tool that has been used in psycho-oncologic research worldwide to detect clinically significant levels of distress in patients with cancer (Donovan et al., 2014). The DT consists of a single item that assesses the global level of distress that has been experienced in the past week, including the present day (Gessler et al., 2008). The scale ranges from 0 (*no distress*) to 10 (*extreme distress*) with a cutoff score of 5, indicating a clinically significant level of distress. Moreover, a DT score of ≥8 has been used in several studies to identify severe distress (Mitchell et al., 2011; Meggiolaro et al., 2016). Furthermore, the DT contains a standardized problem checklist with 34 items answered with “no” or “yes.” These 34 problems refer to five problem areas: practical (5 items, e.g., “transportation”), family (2 items, e.g., “problems with the partner”), emotional (5 items, e.g., “fears”), spiritual (2 items, e.g., “loss of faith”), and physical problems (20 items, e.g., “fatigue”). The DT has been validated in cancer patients with different diagnoses and disease stages (Donovan et al., 2014).

### Statistical Analysis

Data were prepared with *R* in version 3.6.2 (R Core Team, 2018). Statistical analyses were performed using *IBM SPSS Statistics 26.* Frequencies and percentages of patients with values above the DT cutoff score were calculated for the total sample as well as for males and females separately. Moreover, prevalence rates for severe distress (DT score ≥ 8) for the total sample and both sexes were calculated. To assess differences in sociodemographic and medical data regarding the distress level, one-way analyses of variance (ANOVAs) with subsequent *post hoc* tests were conducted. Independent *t*-tests were computed to examine group differences in the DT scores between men and women. Patients were assigned to three age categories (<50, 50–64, and ≥65 years). These age categories were formed as in the study of Meeker et al. (2017) and should allow the comparison of younger patients (< 50 years) who are most likely to be employed and involved in childcare of younger or middle-aged children, with middle-aged patients (50–64 years) who are in an advanced work and family situation, and older, retired patients (≥ 65 years). Age groups were compared regarding the distress level with a one-way ANOVA. Frequencies and percentages of experienced problems were calculated for the total sample and the sexes. Differences in the problem areas between the sexes were tested with independent *t*-tests. Pearson correlations were performed to analyze associations between variables. To investigate the effect of one or more factors on the dependent variable, an analysis of covariance was conducted. To account for the alpha error accumulation, alpha was adjusted to *p* = 0.010 in this case. One-way ANOVAs were used to find differences between the three age categories in the different problem areas. ORs were calculated for the three most prevalent problems and occurrences of high distress.

## Results

### Prevalence of Distress—Total Sample

**FIGURE 1:**
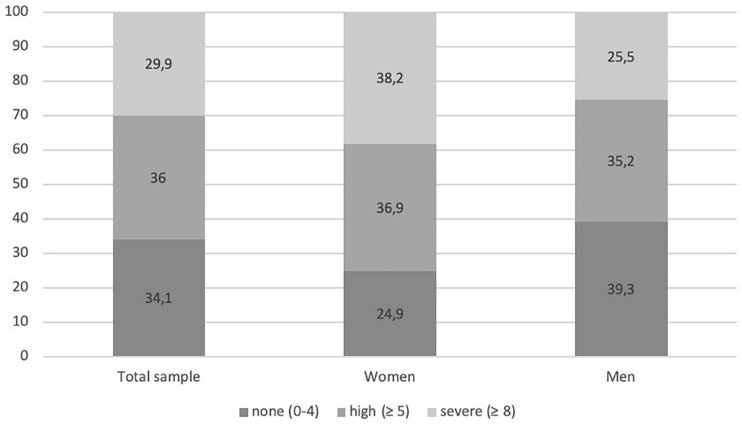
Frequency (%) of distress level measured with the Distress Thermometer in the total sample and for female and male patients.

In the total sample, *n* = 1,735 patients reported a distress value. Among these patients, 65.9% (*n* = 1,143) reported high levels of distress (cutoff value ≥ 5). Moreover, 29.9% (*n* = 519) reported a severe distress value of ≥8 (see Figure 1). There was no significant difference regarding family status, *F*(3, 1,715) = 1.87, *p* = 0.417, but in terms of employment status, *F*(3, 1,588) = 6.23, *p* < 0.001. The Tukey HSD *post-hoc* test revealed significant differences between retired and employed work status with employed patients (*M* = 5.77, *SD* = 2.71) being significantly more distressed than retired patients (*M* = 5.23, *SD* = 2.90). As it is assumed that retired people are older than those who work, an analysis of covariance with age as covariate was calculated. Adjusting for this covariate resulted in a non-significant effect of the between-subject factor work situation. Moreover, patients with children (*M* = 5.46, *SD* = 2.86) were significantly less distressed than patients without children (*M* = 5.93, *SD* = 2.84; *t* = 2.56, *df* = 1,653, *p* = 0.011).

**FIGURE 2:**
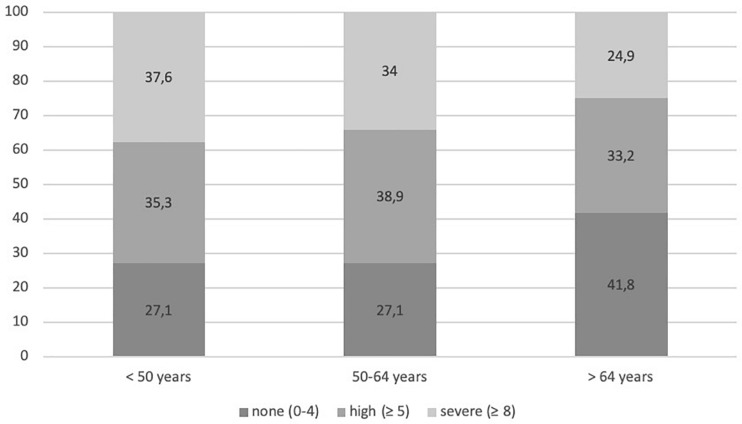
Frequency (%) of distress level measured with the Distress Thermometer in the age groups (<50, 50–64, and >64 years).

A younger age was significantly correlated with higher distress (*r* = -0.15, *p* < 0.001). With regard to differences between age groups, the ANOVA revealed a significant effect, *F*(2, 1,652) = 24.36, *p* < 0.001. *Post-hoc* tests showed that patients below the age of 50 years (*M* = 5.97, *SD* = 2.80) and patients between 50 and 64 years (*M* = 6.00, *SD* = 2.66) were significantly more distressed than patients ≥65 years (*M* = 5.02, *SD* = 2.93; see Figure 2).

Regarding different cancer types, the ANOVA revealed a significant effect, *F*(3, 1,542) = 12.93, *p* < 0.001. *Post-hoc* tests showed that patients with visceral cancer (*M* = 6.03, *SD* = 2.78) were significantly more distressed than patients with urological cancer (*M* = 5.09, SD = 3.04) and skin cancer (*M* = 5.09, *SD* = 2.82). Patients with head and neck cancer (*M* = 5.85, *SD* = 2.51) were also significantly more distressed than patients with urological (*M* = 5.09, *SD* = 3.04) and skin cancer (*M* = 5.09, *SD* = 2.82). There were no age differences between the four cancer groups. Patients with two or more cancer diagnoses (*M* = 5.85, *SD* = 2.78) compared with patients with only one cancer diagnosis (*M* = 5.20, *SD* = 2.88) were significantly more distressed (*t* = -4.71, *df* = 1,658, *p* < 0.001). Moreover, a significant correlation between the number of diagnoses and distress was found (*r* = 0.088, *p* < 0.001).

Regarding the UICC stages, a significant effect was found, *F*(3, 1,150) = 2.94, *p* = 0.032. *Post-hoc* tests revealed a significant difference between stage 3 (*M* = 5.30, *SD* = 2.79) and stage 4 (*M* = 5.96, *SD* = 2.87). Due to the significant age difference between stage 1 and stage 2, a one-way between-subjects analysis of covariance was carried out to assess the impact of age on distress level. The covariate comprised age, and this was significantly related to distress level: *F*(1, 1,127) = 25.57, *p* < 0.001. Adjusting for this covariate resulted in a significant effect of the between-subject factor UICC stage: *F*(3, 1,127) = 3.85, *p* = 0.009. The adjusted distress level for stage 1 was 5.31, stage 2 5.68, stage 3 5.27, and stage 4 5.99. The time since diagnosis in months was not significantly correlated with distress.

### Prevalence of Distress—Sex Differences

Compared with 60.6% (*n* = 673) of men, *N* = 458 (75.1%) of women reported high levels of distress (cutoff ≥ 5). In addition, 38.2% (*n* = 233) of female patients and 25.4% (*n* = 282) of male patients reported severe distress (≥8). Women (*M* = 6.21, *SD* = 2.69) were significantly more distressed than men (*M* = 5.16, *SD* = 2.86; *t* = 7.50, *df* = 1,718, *p* < 0.001; see Figure 1). Due to the significant age difference between men and women, a one-way between-subjects analysis of covariance was carried out to assess the impact of age on distress level. The covariate comprised age, and this was significantly related to distress level: *F*(1, 1,627) = 29.2, *p* < 0.001. Adjusting for this covariate resulted in a significant effect of the between-subject factor sex: *F*(1, 1,627) = 42.9, *p* < 0.001. The adjusted distress levels for females were 6.18 and for males 5.22.

For women, employment status and family status had no effect on the distress level. Regarding cancer type, a significant effect, *F*(3, 499) = 4.37, *p* = 0.005, was found with higher distress in female patients with visceral cancer (*M* = 6.67, *SD* = 2.70) than women with skin cancer (*M* = 5.77, *SD* = 2.74). Regarding age, women between 50 and 64 years (*M* = 6.59, *SD* = 2.41) reported significantly higher distress levels than women aged 65 years and older (*M* = 5.84, *SD* = 2.82) [*F*(2, 570) = 4.52, *p* = 0.011].

For men, employed male patients (*M* = 5.54, *SD* = 2.72) were significantly more distressed than retired men (*M* = 4.79, *SD* = 2.89) [*F*(3, 1,015) = 5.60, *p* = 0.001]. Due to the significant age difference between employed and retired men, a one-way between-subjects analysis of covariance was carried out to assess the impact of age on distress level. The covariate comprised age, and this was significantly related to distress level: *F*(1, 1,042) = 9.16, *p* < 0.01. Adjusting for this covariate resulted in a significant effect of the between-subject factor employment status: *F*(4, 1,042) = 2.37, *p* = 0.05. The adjusted distress levels for retired were 5.04 and for employed males 5.24. Moreover, a significant effect for cancer type, *F*(3, 1,033) = 7.50, *p* < 0.001, was found: men with visceral cancer (*M* = 5.67, *SD* = 2.76) were significantly more distressed than men with skin cancer (*M* = 4.60, *SD* = 2.78) and urological cancer (*M* = 5.01, *SD* = 3.00). Regarding age, a significant effect was found, *F*(2, 1,072) = 14.76, *p* < 0.001: Men younger than 65 years (*M* = 5.71, *SD* = 2.76) and between 50 and 64 years (*M* = 5.62, *SD* = 2.74) were more distressed when compared with men aged 65 years and older (*M* = 4.71, *SD* = 2.90).

### Prevalence of Problems—Sex and Age Differences

As shown in Table 2, the most prevalent problems were emotional problems such as fears (50.1%) and worry (49.9%) and physical problems such as fatigue (49.1%), sleep problems (48.2%), and pain (45.9%). Those five problems were also the most frequently reported problems in both females and males. On average, patients reported having, on average, 6.45 (*SD* = 5.10; range 0–28) of the given 34 problems.

**TABLE 2:** Frequency of cancer and treatment-related problems (DT) of the total sample (*N* = 1,869) and for males (*n* = 1,187) and females (*n* = 666) separately.

	Total sample		Women		Men	
Problems	*n*	%	*n*	%	*n*	%
Fears	875	50.1	408	65.6	458	41.2
Worry	853	49.9	364	61.0	478	43.5
Fatigue	874	49.1	394	63.0	471	41.3
Sleep	851	48.2	357	57.4	485	42.9
Pain	810	45.9	308	50.5	494	43.3
Getting around	791	44.7	289	47.1	492	43.1
Nervousness	589	34.9	247	42.0	340	31.3
Sadness	579	34.3	290	48.9	284	26.2
Eating	478	27.5	216	35.4	260	23.3
Skin dry/itchy	427	24.6	178	29.0	244	21.9
Indigestion	417	24.1	197	32.2	217	19.6
Bathing/dressing	405	23.2	153	25.4	248	22.0
Changes is urination	351	20.4	84	14.2	264	23.7
Tingling in hands/feet	339	19.5	117	19.1	217	19.5
Breathing	319	18.4	131	21.8	182	16.3
Nausea	308	17.8	154	25.7	149	13.3
Constipation	284	16.5	121	19.9	162	14.7
Appearance	267	15.8	116	19.9	150	13.7
Nose dry/congested	255	14.8	112	18.6	140	12.6
Diarrhea	244	14.2	122	20.4	119	10.7
Depression	230	14.0	103	18.5	124	11.6
Feeling swollen	239	14.0	98	16.4	138	12.6
Sexual problems	215	12.9	40	6.9	169	15.8
Mouth sores	212	12.2	104	17.1	107	9.6
Transportation	119	6.9	52	8.7	66	6.0
Housing	118	6.6	58	9.3	59	5.2
Family problems/partner	108	6.3	44	7.3	60	5.4
Family problems/children	92	5.5	43	7.3	47	4.4
Work/school	93	5.5	40	8.7	52	4.8
Fevers	90	5.2	36	6.1	53	4.7
Insurance	69	4.0	19	3.1	49	4.4
Loss of faith	65	3.9	18	3.1	46	4.4
Spiritual/religious concerns relating to God	56	3.4	28	4.8	28	2.6
Child care	28	1.7	16	2.7	11	1.0

**Sample sizes for the different problems vary between N = 1,644 and N = 1,781.**

With reference to the different problem areas (practical, family, emotional, spiritual, and physical problems; Table 3), women (*M* = 2.19, *SD* = 1.59) had significantly more problems in the emotional problem area, compared with men (*M* = 1.48, *SD* = 1.59; *t* = 9.12, *df* = 1,784, *p* < 0.001), in physical problems: women (*M* = 5.11, *SD* = 3.88) reported significantly more problems than men (*M* = 4.03, *SD* = 3.68; *t* = 5.92, *df* = 1,831, *p* < 0.001) and practical problems (women: *M* = 0.29, *SD* = 0.60; men: *M* = 0.21, *SD* = 0.55; *t* = 2.94, *df* = 1,211.96, *p* = 0.003).

**TABLE 3:** Differences between males and females regarding the different problem areas of the problem checklist.

	Women		Men					
Problem Areas	*M*	*SD*	*M*	*SD*	*t*	*df*	*p*^*a*^	*d*
Practical problems	0.29	0.60	0.21	0.55	2.94	1,211.96	0.003	0.17
Family problems	0.14	0.42	0.09	0.36	2.31	1,088.55	0.015	0.14
Emotional problems	2.19	1.59	1.48	1.59	9.12	1,784	<0.001	0.43
Spiritual problems	0.08	0.31	0.07	0.31	0.48	1,664	0.629	0.02
Physical problems	5.11	3.88	4.03	3.68	5.92	1,831	<0.001	0.28

**^*a*^Significance level p = 0.010.**

When comparing the three different age groups, a significant effect was found for practical problems [*F*(2, 1,715) = 20.37, *p* < 0.001], family problems [*F*(2, 1,675) = 7.13, *p* = 0.001], and emotional problems [*F*(2, 1,716) = 54.40, *p* < 0.001]. Regarding practical problems, patients under the age of 50 years (*M* = 0.38, *SD* = 0.74) reported significantly more practical problems than patients between 50 and 64 years (*M* = 0.27, *SD* = 0.59) and patients ≥65 years (*M* = 0.15, *SD* = 0.44). Patients aged 50–64 years reported significantly more practical problems than patients aged 65 years and older. Patients ≥65 years (*M* = 0.07, *SD* = 0.31) reported significantly fewer family problems compared with middle-aged patients (*M* = 0.13, *SD* = 0.41) and younger patients (*M* = 0.16, *SD* = 0.47). Younger patients (<50 years) (*M* = 2.12, *SD* = 1.62), as well as middle-aged patients (50–64 years) (*M* = 2.08, *SD* = 1.65), experienced significantly more emotional problems than older patients (≥65 years) (*M* = 1.30, *SD* = 1.48).

### Association of High Distress and the Most Common Problems

As shown in Table 4, patients who reported any one of the three most frequently reported problems (fears, worry, and fatigue) have a risk more than three times as high to be highly distressed than those who do not experience any of these problems (OR 3.51, 95% confidence interval: 2.67, 4.62). Those who exclusively experienced fears, worry, or fatigue had more than five to seven times higher chance of being highly distressed than those who did not. For patients reporting all three problems, the OR of high distress was 23.90 (95% confidence interval: 16.07, 35.53), increasing their chance for high levels of distress by a factor of almost 24 compared with those without these problems.

**TABLE 4:** Odds ratios for high distress for the three most frequently reported problems.

		High distress (DT)		
Variable	*n*	%	OR	95% CI
Fears	713	84.78	6.84	[5.41, 8.65]
Worry	682	83.48	6.15	[4.88, 7.75]
Fatigue	683	83.39	5.34	[4.25, 6.71]
Any 1 of the 3 problems^*a*^	261	63.66	3.51	[2.67, 4.62]
Any 2 of the 3 problems^*b*^	301	80.27	8.16	[5.95, 11.19]
All 3 problems	405	92.26	23.90	[16.07, 35.53]

**^*a*^Any 1 problem out of fatigue, worry, or fears has been reported. ^*b*^Any 2 problems out of fatigue, worry, or fears have been reported. DT, Distress Thermometer.**

## Discussion

This study aimed to investigate the prevalence of high distress in a large sample of cancer inpatients and to identify risk factors most strongly associated with high psychological distress, as well as the prevalence of common problems experienced by cancer patients.

### Prevalence of High Distress

In this sample, 65.9% of patients experienced high levels of distress, with 75.1% of women and 60.6% of men scoring above the cutoff value. Of the total sample, 29.9% reported severe levels of distress (≥8). Women scored even higher with 38.2% (men: 25.4%). Compared with the results of Mehnert et al. (2018), with 52% of the German sample (in- and outpatients) experienced high distress (women: 56.8%, men: 46.7%), the present results are higher for the total sample and both sexes as well. Compared with the studies of Zabora et al. (2001) and Kendall et al. (2011) (32 and 35.1%) who investigated only outpatients, the prevalence of distress found in this sample is markedly higher. Clark et al. (2011), on the other hand, who examined also inpatients, found a similar distress prevalence of 63.5%. Regarding the prevalence of severe distress, other studies with mixed samples of cancer patients as well found lower prevalence rates, for example, Meggiolaro et al. (2016) 14.9% or Herschbach et al. (2019) 12.7%.

Explanatory factors for the higher prevalence found in this study with cancer inpatients might be stressful events such as diagnosis and intense treatment that usually takes place during a hospital stay. This might increase the risk of distress and other mental disorder in inpatients compared with outpatients. Additionally, inpatients were shown to feel less control over their symptoms, the course of illness, and over medical care and treatment (Peters and Sellick, 2006). Perceived uncertainty regarding the diagnosis, the symptoms, and their seriousness, as well as the fear of pain and discomfort as a result of the treatment, might also be more serious in hospitalized patients (Mishel, 1984). Mishel (1984) also assumed that trying to understand the medical jargon, the lack of clear communication, and limited comprehension of events is another major source of stress resulting from hospitalization. All these factors might contribute to inpatients experiencing more psychological distress than outpatients. A further explanation might be that inpatients generally have a poorer physical health status with higher levels of symptom severity and, therefore, higher prevalence of psychological distress (Peters and Sellick, 2006). Hinz et al. (2018) found that inpatients with cancer showed the lowest levels of the general quality of life when compared with cancer outpatients and patients from rehabilitative settings. A decreased ability to carry out daily activities might be another contributing factor as inpatients are separated from their spouses, families, and familiar surroundings (Peters and Sellick, 2006). A further explanation could be that the patients have not yet received a psycho-oncological intervention or support, especially as there is, on average, less than 6 months between the date of initial diagnosis and screening for distress. A psycho-oncological intervention can significantly reduce distress (Blenkiron et al., 2014). Outpatients and especially patients from rehabilitative settings might be more likely to have already received an intervention compared with inpatients. The findings suggest that inpatients are generally exposed to more factors that might increase the risk of psychological distress than outpatients and patients in rehabilitative care settings.

### Risk Factors for High Distress and Common Problems

Several risk factors for distress were identified. The female sex and stages 4 of cancer were significantly related to high levels of distress. Moreover, patients with more than one cancer diagnosis are more distressed than patients with only one cancer diagnosis. These results are in line with other studies identifying women as well as an advanced stage of the disease as a risk factor for high levels of distress (Giese-Davis et al., 2012; Kim et al., 2017; Hamilton and Kroska, 2018; Mehnert et al., 2018; Weis et al., 2018).

Regarding cancer type, this study found patients with visceral cancer and head and neck cancer to be significantly more distressed than patients with skin and urological cancer. Linden et al. (2012) also found patients with skin and prostate cancer to be less emotionally distressed than the average cancer patient. In the sample of Carlson et al. (2019) with more than 4,000 patients, men with prostate cancer and women with lung, head and neck, and pancreatic cancer were more likely to be distressed than patients with other cancer types. Although differences in distress in several tumor entities have so far been less the subject of studies, some reasons seem possible why some cancers are associated with more distress than others. Some types of cancer require more intensive treatment, have a poorer prognosis, or more serious consequences. However, it cannot automatically be assumed that this is also associated with higher psychological distress, as other factors such as their own coping factors or social support can also be important influencing factors.

Moreover, women were significantly more distressed than men and reported more practical, emotional, and physical problems in the problem checklist than men. One explanation might be the increased use of an emotional coping style by women. Emotion-focused coping is positively associated with distress (Matud et al., 2015). Keller and Henrich (1999) suggest that women are more likely than men to engage in illness behavior. Women might adopt the sick-role more easily, and their awareness of one’s distress might help them to communicate their symptoms and distress more easily, whereas men might tend to withhold or underreport their symptoms (Keller and Henrich, 1999). Mehnert et al. (2018) suggest three further explanations for higher levels of psychological distress in women: First, women might be more emotionally expressive than men due to their socialization. Second, there is the assumption that women might face more (severe, persistent) stressors than men. Third, women might lack sufficient or less effective coping resources/strategies for handling the stressor they were exposed to than men (Thoits, 1991).

Compared with older patients (≥65 years), younger patients and middle-aged (50–64 years) patients were significantly more distressed. This result is in line with the results of Burgoyne et al. (2015), who found that younger patients with cancer (18–39 years) reported higher cancer-related distress than older patients (65–90 years) but similar distress levels when compared with middle-aged patients (40–64 years). Carlson et al. (2019) also found that patients aged between 30 and 69 years were more distressed than those aged 70 years and older. Other studies found a general decrease in distress with advanced age in patients with cancer (Baider et al., 2003; Jorm et al., 2005).

A further result was that younger patients (<50 years) reported the most practical problems (e.g., problems with childcare, money, or work) followed by middle-aged patients (50–64 years). Older patients (≥65 years) reported significantly less practical and family problems. One possible explanation might be that younger patients experience higher levels of disruption of everyday routines (Mor et al., 1994). Younger patients with cancer usually have multiple responsibilities regarding childcare, work, and other social role demands as well as a spouse who might be engaged in a full-time job and, therefore, might not be able to provide sufficient social support. Consequently, they experience more competing demands in their stage of life and therefore experience bigger disruptions of normal routines as a consequence of the disease (Mor et al., 1994). Older patients, on the other hand, might have fewer competing demands in their stage of life. Especially patients older than 65 years are often retired and thus often in a safer economic situation. The demands of parenting are on a low level due to the older age of the children for middle-aged and especially older patients. The spouse might no longer be engaged in full-time employment and can, therefore, provide sufficient social support and practical assistance. Disruptions in practical areas such as childcare, finances, or work are less prevalent and intense among the older age group (Mor et al., 1994). Moreover, younger patients might experience greater illness intrusiveness, which assesses the degree to which the cancer diagnosis and treatment interfere with different life areas (Avis et al., 2012).

Moreover, the study results suggest that younger patients and middle-aged patients reported significantly more emotional problems than patients above the age of 65 years. Mor et al. (1994) found that younger patients with cancer reported higher levels of negative affect than older patients and that older age was predictive for enhanced emotional well-being in cancer patients. Another explanation might be that older patients with cancer tend to be more emotionally resilient because they might already have been exposed to various serious stressors during their course of life. Therefore, they might possess more experience and coping resources in dealing with the diagnosis and treatment of a disease such as cancer (Sammarco, 2009; Levkovich et al., 2018). Moreover, especially younger patients who are fully engaged in their lives might perceive their illness as less fair and devastating for their future, which could cause more emotional distress among them (Mor et al., 1994).

### Prevalence of Problems

The most prevalent problems experienced by almost half of the sample were fears, worry, and fatigue. For women, the percentages for fear, worry, and fatigue were 10–15% higher as in the total sample. Patients who reported fear, worry, or fatigue had a more than five to seven times higher chance of being highly distressed. For patients who reported all three of the most prevalent problems, the odds of experiencing high levels of distress were almost 24 times higher than that of those who reported none of these problems. The results illustrate the high prevalence of specific emotional and physical problems such as fears, worry, and fatigue among patients with cancer and the high risk for psychological distress when experiencing them. Moreover, these results replicate other studies’ findings (Lee et al., 2010; Kendall et al., 2011; Blenkiron et al., 2014). In the study of Mehnert et al. (2018), fatigue and sleep problems were the most prevalent physical problems, and worry and fears were the most prevalent emotional problems. Mehnert et al. (2018) suggest that these particular problems might function as “red flags” (p. 80) in the routine distress screening for cancer patients. Clinicians might rapidly identify patients who have a high risk of experiencing psychological distress when they regularly check for these particular problems and might, therefore, be able to provide appropriate psycho-oncological interventions (Bultz and Johansen, 2011).

Nevertheless, despite the high prevalence of distress among cancer patients and the benefit from a psycho-oncological intervention, a high percentage of distressed cancer patients do not want or eventually do not use psychosocial support (Pichler et al., 2019). Pichler et al. (2019) found that in a sample of 925 German cancer inpatients, 71.6% declined psychological support. Among those patients experiencing high levels of distress (46.2%), 53.9% declined psychological support. Future research should address this problem more closely and investigate possible relationships between the decline of psychosocial support and factors within the patient and external factors. This way, patients at high risk of distress who decline psychosocial support might be more easily identified.

### Limitations and Further Directions

There are some limitations to this study. First, because of the cross-sectional design of this study, causality regarding associations cannot be inferred. Future studies should measure psychological distress in cancer patients at several time points to assess changes in the disease status. Furthermore, psychological distress was assessed using the DT. Especially in those identified as having high psychological distress, a full mental status assessment would be important to provide patient-tailored intervention (Mehnert et al., 2018). Finally, the frequency of cancer types varied due to the mixed cancer sample. The selectivity of the sample might make it difficult to generalize the results across cancer patients in general, as distributions of the different cancer entities were not representing the general population of patients with cancer. Future research should investigate larger and more representative samples of different cancer types.

### Practical Implications

The results suggest that there is a high percentage of cancer patients that experience high levels of distress. Especially emotional and physical problems are highly common among cancer patients. Moreover, several risk factors for high psychological distress were identified. These results can help to identify patients with clinically significant levels of distress and therefore offer more distressed patients with psychosocial and psycho-oncological support. Given the high prevalence of distress found in this sample, clinicians should not only routinely screen for distress but should also pay special attention to certain problem fields that are highly indicative of psychological distress. The results might contribute to a better understanding of psychological distress in patients with cancer and could, therefore, optimize the identification and treatment of these patients.

## Data Availability Statement

The raw data supporting the conclusions of this article will be made available by the authors, without undue reservation.

## Ethics Statement

Ethical review and approval was not required for the study on human participants in accordance with the local legislation and institutional requirements. Written informed consent for participation was not required for this study in accordance with the national legislation and the institutional requirements.

## Author Contributions

TZ designed the study. LP wrote the first draft and contributed to the data analysis. JB prepared and analyzed the data. All authors contributed significantly to the interpretation of the data and the final version of the manuscript and gave final approval of the version to be published.

## Conflict of Interest

The authors declare that the research was conducted in the absence of any commercial or financial relationships that could be construed as a potential conflict of interest.
